# Simple Estimation of Incident HIV Infection Rates in Notification Cohorts Based on Window Periods of Algorithms for Evaluation of Line-Immunoassay Result Patterns

**DOI:** 10.1371/journal.pone.0071662

**Published:** 2013-08-26

**Authors:** Jörg Schüpbach, Martin D. Gebhardt, Alexandra U. Scherrer, Leslie R. Bisset, Christoph Niederhauser, Stephan Regenass, Sabine Yerly, Vincent Aubert, Franziska Suter, Stefan Pfister, Gladys Martinetti, Corinne Andreutti, Thomas Klimkait, Marcel Brandenberger, Huldrych F. Günthard, the Swiss HIV Cohort Study

**Affiliations:** 1 University of Zurich, Institute of Medical Virology, Swiss National Center for Retroviruses, Zurich, Switzerland; 2 Swiss Federal Office of Public Health, Berne, Switzerland; 3 Division of Infectious Diseases and Hospital Epidemiology, University Hospital Zurich, University of Zurich, Zurich, Switzerland; 4 Blood Transfusion Service, Swiss Red Cross Berne, Berne, Switzerland; 5 University Hospital, Clinic for Immunology, Zurich, Switzerland; 6 Geneva University Hospitals, Laboratory of Virology, Genève, Switzerland; 7 University Hospital, Service of Immunology and Allergy, University Hospital Center, Lausanne, Switzerland; 8 University of Berne, Institute of Infectious Diseases, Berne, Switzerland; 9 Institut Dr. Viollier AG, Basel, Switzerland; 10 Ente ospedaliero cantonale, Servizio di microbiologia, Bellinzona, Switzerland; 11 Clinique de la Source, Laboratoire, Lausanne, Switzerland; 12 University of Basel, Institute for Medical Microbiology, Basel, Switzerland; 13 Labor Synlab Luzern, Lucerne, Switzerland; Chinese Academy of Sciences, Wuhan Institute of Virology, China

## Abstract

**Background:**

Tests for recent infections (TRIs) are important for HIV surveillance. We have shown that a patient's antibody pattern in a confirmatory line immunoassay (Inno-Lia) also yields information on time since infection. We have published algorithms which, with a certain sensitivity and specificity, distinguish between incident (< = 12 months) and older infection. In order to use these algorithms like other TRIs, i.e., based on their windows, we now determined their window periods.

**Methods:**

We classified Inno-Lia results of 527 treatment-naïve patients with HIV-1 infection < = 12 months according to incidence by 25 algorithms. The time after which all infections were ruled older, i.e. the algorithm's window, was determined by linear regression of the proportion ruled incident in dependence of time since infection. Window-based incident infection rates (IIR) were determined utilizing the relationship ‘Prevalence  =  Incidence x Duration’ in four annual cohorts of HIV-1 notifications. Results were compared to performance-based IIR also derived from Inno-Lia results, but utilizing the relationship ‘incident  =  true incident + false incident’ and also to the IIR derived from the BED incidence assay.

**Results:**

Window periods varied between 45.8 and 130.1 days and correlated well with the algorithms' diagnostic sensitivity (R^2^ = 0.962; P<0.0001). Among the 25 algorithms, the mean window-based IIR among the 748 notifications of 2005/06 was 0.457 compared to 0.453 obtained for performance-based IIR with a model not correcting for selection bias. Evaluation of BED results using a window of 153 days yielded an IIR of 0.669. Window-based IIR and performance-based IIR increased by 22.4% and respectively 30.6% in 2008, while 2009 and 2010 showed a return to baseline for both methods.

**Conclusions:**

IIR estimations by window- and performance-based evaluations of Inno-Lia algorithm results were similar and can be used together to assess IIR changes between annual HIV notification cohorts.

## Introduction

Information on the incidence of HIV infection is crucial for monitoring the dynamics of the HIV epidemic in affected countries. Therefore, ‘serologic testing algorithms for recent HIV seroconversion’ (STARHS) [1]–[4], now also more generally called ‘tests for recent infections’ (TRIs) or ‘recent infection testing algorithms’ (RITA) have been developed [5], [6]. STARHS make use of the fact that the HIV antibody response evolves during the first few months of infection with respect to concentration [7]–[9], proportion of total IgG [10], isotype [11] or avidity [12]. Some more recently developed TRIs are based on the genomic diversity evolving in an infected individual [13]–[15]. The time during which these properties remain below a predetermined cutoff may greatly differ individually, and its mean duration or ‘window-period’ has to be established by testing specimens from individuals with a known date of HIV seroconversion [16]. Estimation of the incidence in a population is based on the relationship ‘Prevalence  =  Incidence x Duration’ [4], [5].

STARHS require a special assay of reduced analytical sensitivity; hence they are also called ‘detuned’ assays. The reduced sensitivity renders these tests unsuitable for the diagnosis of HIV infection and restricts their use to epidemiological studies. In contrast, we have shown that a patient's antibody reaction in a widely used confirmatory line immunoassay, the Inno-Lia^TM^ HIV I/II Score (Inno-Lia), provides information on the duration of infection similar to that of a commercial enzyme immunoassay (EIA), the so-called BED incidence EIA [10], [17]. The Inno-Lia is a type of second-generation Western blot (WB) that measures antibodies to different HIV antigens in a semi-quantitative way. As both the pattern and intensity of HIV-specific antibodies evolve during the first weeks to months after infection, it is possible to define algorithms (Alg) which, with a certain diagnostic sensitivity and specificity, differentiate between early and late antibody patterns. If the diagnostic sensitivity and specificity of an algorithm are known, which requires prior testing of suitable reference groups of infections of either less or more than 12 months duration, it is possible to estimate the incidence by means of the basic diagnostic rule ‘n_tested incident_  = n_true incident_ +n_false incident_’, whereby true-incident and false-incident are calculated based on the pre-determined values for diagnostic sensitivity and specificity [17].

In previous work, we have determined the diagnostic sensitivity and specificity of more than 20 different Inno-Lia algorithms for differentiating between HIV-1 infections of less or more than 12 months duration. A study of 714 patients selected from the Swiss HIV Cohort Study (SHCS), who had been infected for at least 12 months and represented all clinical stages and major clades of HIV-1, showed that none of these variables affected the incident infection algorithms [18]. Of the 714 patients investigated in that study, only 94 were infected by HIV-1 subtype B, while 620 patients were infected by one of 15 different non-B clades. The study showed that none of these non-B clades impaired the diagnostic specificity of the method in comparison to subtype B. Although a viral RNA load below 50 copies/mL significantly reduced the specificity among patients receiving antiretroviral treatment (ART), age was the sole factor which weakly impaired the test specificity in untreated patients [18]. In another study, we assessed the diagnostic performance of the algorithms based on 527 incident and 740 older infections. The ten best-performing algorithms had an unadjusted mean sensitivity of 59.4% to recognize infections of up to 12 months duration and a mean specificity of 95.1% among patients infected for longer than 12 months. Using these ten algorithms in combination, we were able to identify distinct changes between the incident infection rates (IIR) of four successive annual cohorts of HIV-1 notifications [19].

The present study now explores the option to utilize the Inno-Lia algorithms in the same way as other TRIs, i.e. based on their window periods. We determined the window periods of all hitherto published Inno-Lia algorithms and compared window-based and performance-based incident infection rates in the four previously studied annual cohorts of HIV-1 notifications [19].

## Methods

### Ethics statement

The present study investigated patients of the Zurich Primary HIV Infection (ZPHI) study [20], [21] and data from anonymized HIV notifications to the Swiss Federal Office of Public Health (SFOPH). The ZPHI study was approved by the ethical committee of the Zurich University Hospital, and all participating patients gave their written informed consent to the study goals. No informed consent was needed for the anonymized notifications of newly diagnosed HIV infections to the SFOPH, as these are required by federal legislation.

### Patients and specimens

In order to enable an optimal comparison of window-based and diagnostic-performance-based estimation of the incident infection rate (IIR), the patients and specimens were exactly as used in a previous study [19]. For determination of the window periods of the Inno-Lia algorithms, we used a group of 527 patients with HIV-1 infection of up to 12.0 months duration ( =  incident infection). In short, 144 of the 527 patients originated from the ZPHI study, while the remaining 383 patients were identified among the anonymized HIV-1 notifications received by the SFOPH from April 2007 to December 2010.

The ZPHI study is an observational, open label, nonrandomized, single-center study (ClinicalTrials.gov identification no. NCT00537966) [21]. Patients with acute or recent HIV-1 infection were included. Acute HIV-1 infection was defined as 1) presentation of the acute retroviral syndrome (ARS) and a negative or indeterminate WB or Inno-Lia results in the presence of a positive p24 antigen test and/or a detectable viral load; or 2) a documented seroconversion with or without symptoms no more than 90 days ago. Recent infection in the context of the ZPHI study was defined as 3) a possible ARS, a positive WB or Inno-Lia result, detectable viral load, and a positive HIV gp120 avidity respectively detuned assay result [22]; or 4) a documented acute HIV-1 infection with referral to our center within 90 days after estimated date of infection (EDI). For each patient, EDI was determined by taking into account the pattern of different assay results (first positive and last negative HIV-test; negative, indeterminate and positive WB; positive p24 Ag; antibody avidity assay), patient's reports of unambiguous risk contacts, and timing of onset of ARS symptoms. With respect to WB results, the following rules were applied to determine the EDI: (i) Negative WB (Fiebig stages I-III) [23]: If a single risk contact was reported within the last three weeks before the date of WB, this date was taken as EDI. In contrast, if no history of risk contacts was reported, infection was assumed to have occurred 14 days before the WB date. (ii) Indeterminate WB (Fiebig stage IV): If a single risk contact was reported between 2 and 6 weeks before the date of WB, this date was taken as EDI. In case of several risk contacts, a higher and lower range was estimated and the mean of this range was taken as EDI. (iii) Positive WB (Fiebig stages V–VI): If a single risk contact occurred 6 weeks or earlier before the date of the WB, this date was taken as EDI if seroconversion was documented. If a seroconversion within 6 months was clearly documented without history of risk contact, the mean date between the two tests (last negative and first positive HIV-test) was taken as EDI. If a patient had a history of an ARS, a fully converted WB, but no documented seroconversion and a negative detuned or avidity assay, the EDI was defined as the date 20 days before the onset of the ARS. These EDI definitions have been successfully used and validated in previous publications [13], [21], [24], [25].

Incident infection among the HIV notifications to the SFOPH was identified exactly as published previously [19], i.e. as a case that met one of the following definitions. (1) Laboratory evidence of seroconversion at the time of diagnosis, i.e., a reactive 4^th^-generation HIV-1/2/O antibody/p24 antigen combination screening test and a positive virus component test (HIV-1 RNA or DNA or p24 antigen) in combination with a negative 3^rd^-generation HIV-1/2/O antibody-only enzyme immunoassay and/or a negative or indeterminate Inno-Lia result according to the manufacturer's instructions for result interpretation; (2) a self-reported or documented negative HIV screening result no more than 12 months before diagnosis; and (3) documented signs of ARS no more than 90 days before diagnosis [26]. EDI among the notifications was defined as 14 days before the reported date of onset of ARS symptoms or the mean date between the last negative and first positive HIV-test.

### Serological differentiation of incident and older HIV-1 infection

Inno-Lia^TM^ HIV I/II Score assay results (Innogenetics, Ghent, Belgium) of all investigated patients as well as the incident infection classifications by 26 published Inno-Lia algorithms (Alg) were available from the above-mentioned study described in detail in reference [19]. The Inno-Lia is a CE-marked, Western blot–like line immunoassay that measures antibodies against recombinant proteins or synthetic peptides of HIV-1 group M, HIV-1 group O, or HIV-2. The antigens are coated as 7 discrete lines on a nylon strip with plastic backing. As each test strip also contains three quantitative internal standards, a semi-quantitative ranking of the different antibody reactions is possible [27] [28]. Antibody reaction to each of the 7 HIV antigen bands present on the test strips (sgp120 [including group O peptides], gp41, p31, p24 and p17 of HIV-1, and sgp105 and gp36 of HIV-2) was assessed either visually or by the automated scanner–based LiRAS system (Innogenetics). Based on the three internal standards, which define reaction levels of 0.5 (+/−), 1 and 3 for each test strip, the antibody reaction to each HIV antigen was classified into one of six possible intensity scores (0, 0.5, 1, 2, 3, or 4). For the present study, only the antibody reaction to HIV-1 was of relevance. Therefore, each patient's pattern of antibodies against the five HIV-1 antigens gp120, gp41, p31, p24 and p17 was subjected to analysis by each of the 26 incident infection algorithms.

### Inno-Lia incident infection algorithms

The 26 algorithms (Algs) for incident HIV-1 infection, all described in Supporting Material S1, were developed empirically by investigating which Inno-Lia antibody patterns were found at maximal frequency in a group of patients with ≤12 months of infection ( = incident infections) and at minimal frequency in a group of patients with >12 months duration of infection, as described in detail in a previous publication [17]. Twelve of the algorithms, Alg2 to Alg13, were published in that paper. The other 14 were developed more recently in the same way and based on the same dataset; they were used in two further studies [18], [19]. All 26 algorithms were applied to the collected Inno-Lia data of the present study. Thus, each Inno-Lia band pattern was classified by 26 algorithms as representing either an incident or older HIV-1 infection.

### Determination of window periods and incident infection rates

The window period of an algorithm was defined as the duration of HIV-1 infection after which, according to that algorithm, all investigated samples would be classified as representing an older infection. This was determined by bivariate plots for each algorithm as follows: Estimated duration of infection (x-axis) was divided into consecutive 2-week intervals. If a 2-week interval contained fewer than 20 data-points, two or more consecutive intervals were pooled until they contained at least 20 data-points. The percentage of samples categorized as incident per total number of samples in each interval (y-axis) was plotted to the midpoint of the respective interval. In a next step, the curves (percentage of incident infection in dependence of time of the various algorithms were inspected in order to identify the time interval in which the regression curve was linear. This time interval included at least four consecutive midpoints. Linear regression was then used in the selected time interval to calculate the time-point and its 95% confidence interval (95% CI) at which 100% of the patients had converted from incident to older infection status (intersection of the regression curve with the x-axis). We also determined the time-points and their 95% CI at which 0% or respectively 50% of the patients had converted to older infection status.

For calculation of the incident infection rate (IIR) in annual cohorts of HIV-1 notifications, i.e., of the proportion of the notified HIV-1 infections that had occurred within the past 12 months, the equation IIR-W  =  n_tested incident_/n_tested_ * 365/window was used, wherein IIR-W is the window-based IIR and *n_tested_* equals the number of annual notifications [5]. The *raw IIR-W* thus obtained for each algorithm was furthermore adjusted for the algorithm's pre-determined diagnostic specificity among patients infected for >12 months (*raw IIR-W* x %Specificity/100) [19]. The IIR derived from BED-EIA results was based on a window of 153 days, as by the manufacturer's instructions.

Performance-based IIR (IIR-P) were calculated based on the relationship n_tested incident_  = n_true- incident_ +n_false- incident_, wherein n_true- incident_  =  n_tested_ × IIR-P ×%Sensitivity/100 and n_false- incident_  = n_tested_ ×(1–IIR-P)×(1−%Specificity/100). Therefore, as published previously [17] [19], IIR-P  =  (n_tested incident_/n_tested_ +%Specificity/100−1)/(%Sensitivity/100+%Specificity/100−1). Three different diagnostic sensitivities, S_1_, S_2_ and S_3_, were used for calculation of IIR-P, as described in detail in [19]. In short, S_1_ averages the diagnostic sensitivities found in that study for each algorithm in the four quarters of the 12-months recent infection period. It corresponds to a model that assumes an even distribution of diagnosing incident infections over all four quarters. This model is probably incorrect, however, as many HIV-exposed individuals will seek early clarification of their HIV status. Sensitivity S_2_ thus accounts for this bias by weighting the number of cases each quarter contributes to the total number of cases. Thus, the adjusted, weighted sensitivities S_2_ were calculated by multiplying the quarter sensitivities used for determination of S_1_ with the percentage of cases a quarter contributed to total cases and then averaging the products. Sensitivity S_3_ further adjusts for bias exerted by symptomatic patients, who are more likely to be diagnosed than asymptomatic individuals. For determination of S_3_ all cases judged incident because of reported signs or symptoms of ARS were excluded and only the notifications with a previous negative HIV test were considered for the calculation of diagnostic sensitivity. In comparison, S_1_ < S_3_ < S_2_, while IIR-P_1_ > IIR-P_3_ > IIR-P_3_ [19].

### Statistics

Frequencies were compared by 2×2 tables, means by paired t-test or Wilcoxon's signed rank test, as indicated in the text; all tests were two-sided. Correlations were assessed by Pearson's test using Fisher's r to z transformation. Statistical analyses were performed either in Excel® or StatView® 5.0 for Macintosh (SAS Institute Inc., Cary, North Carolina, U.S.A.).

## Results

Inno-Lia data from a total of 527 ART-naïve patients in their first year of HIV-1 infection were used for determining the window periods of 26 previously published incident infection algorithms, as described under Methods. The main characteristics of the patients are summarized in Table 1. A majority of the individuals were males. Men who had sex with men (MSM) represented the most frequent transmission risk. Quartiles indicate that three-quarters of the patients had been infected for three months or less.

**Table 1:** Characteristics of the 527 patients with incident HIV-1 infection.

Patient origin, n (%)	ZPHI study	144	(27.3)
	HIV notifications to SFOPH	383	(72.7)
Sex, n (%)	Male	461	(87.5)
	Female	66	(12.5)
Risk, n (%)	MSM	344	(65.3)
	HET	139	(26.4)
	IVDU	21	(4.0)
	OTH	1	(0.2)
	Unknown	22	(4.2)
Age, median (IQR)		35	(29–43)
Months of infection, median (IQR)		1.4	(0.5–3.0)
HIV-1 RNA, median log[copies/mL] (IQR)		5.2	(4.5–6.1)

Abbreviations: SFOPH, Swiss Federal Office of Public Health; MSM, men who have sex with men; HET, heterosexual; IVDU, intravenous drug use; OTH, other; IQR, interquartile range.

### Determination of algorithm window lengths

**Figure 1:**
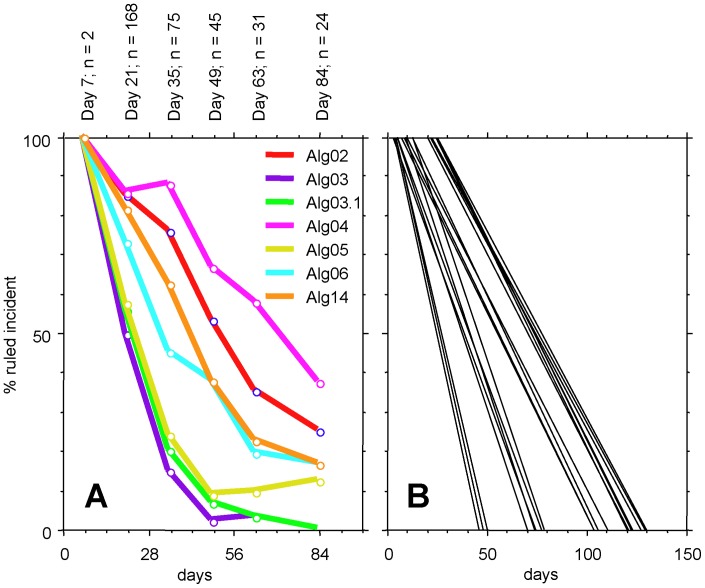
Percentage of cases ruled incident in dependence of time. A. Curves of selected representative algorithms. For algorithm definitions refer to Supporting Information 1. Text on top of the panel denotes the interval midpoints and the number of cases in each interval. B. Linear regression curves of all algorithms except Alg10.

The proportions of cases classified as incident infection by 7 selected algorithms at different time-points are shown in Fig. 1, panel A. The selected curves include six single-band algorithms based on antibody reaction to gp120, gp41, p31, p24, or p17 and one combination algorithm (Alg14; see Supporting Material S1 for the definitions of all algorithms). Conversion from incident to older infection status among these 7 algorithms occurred first for Alg3 (gp41 band ≤0.5), tightly followed by Alg3.1 (gp41≤1) and Alg5 (p24≤0.5), Alg6 (p17≤0.5), Alg14, Alg2 (gp120≤1) and finally Alg4 (p31 = 0). The time intervals in which these curves were considered linear were identified as extending from days 21 to 63 for Algs 3, 3.1 and 5, days 21 to 63 for Alg6 and days 35 to 84 for Algs 14, 2 and 4. The time intervals exhibiting linearity of the curve were established in the same way for all other algorithms. Alg10 (if p31 = 0 AND p24≥2, then incident, else older), designed to increase the long-term specificity when combined with other algorithms, exhibited a tunnel-shaped curve not permitting the determination of a window. The 25 remaining linear regression curves are shown in Fig. 1, panel B. The parameters a and b which, based on the equation y = ax+b, define the linear regression curve are shown in columns C and D of the Supporting Material S2. Likewise, the data-points selected and the squared correlation coefficients R^2^ are listed in columns N to P of that document.

The time-points at which 0%, 50% or 100% of the investigated patients had converted from incident to older infection status, as ruled by a given algorithm, are listed in Table 2. The latest time-points at which all cases were still ruled as incident infection extended from 3.2 days for Alg3 to 25.1 days for Alg4.1. The time-points at which 50% had converted to an interpretation of older infection varied between 24.5 and 77.6 days (same algorithms). Finally, the time-points at which all cases were classified as older infections (full window) extended from 45.8 to 130.1 days (again same algorithms). The 95% confidence intervals (CI) of the full window are also shown in Table 2. Complete data including the 95% CI of the 0% and 50% windows are contained in columns A to P of Supporting Material S2.

**Table 2:** Inno-Lia incident infection algorithms and the estimated time after infection in days at which 0%, 50% or 100% of the patients have converted from incident to older infection status.

Alg #	0% conversion	50% conversion	100% conversion
	mean	mean	mean (95% CI)*
2	6.9	55.3	103.8 (92.0–121.7)
3	3.2	24.5	45.8 (33.0–114.0)
3.1	3.9	26.1	48.2 (36.0–94.0)
3.2	5.7	44.9	95.4 (67.0–85.0)
4	19.9	70.4	121.0 (99.0–177.5)
4.1	25.1	77.6	130.1 (94.0–570.0)
5	4.1	26.9	49.7 (38.0–86.0)
6	3.1	38.7	74.2 (63.0–96.0)
7	12.2	58.8	105.3 (92.0–133.0)
8	9.4	59.9	110.3 (90.0–176.0)
8.1	9.4	59.9	110.3 (90.0–176.0)
9	8.1	55.9	103.6 (80.0–250.0)
11	21.6	74.5	127.4 (109.0–162.0)
11.1	21.6	74.5	127.4 (109.0–162.0)
11.2	21.6	74.5	127.4 (109.0–162.0)
12	22.0	75.6	129.3 (106.0–185.0)
12.1	22.0	75.6	129.3 (106.0–186.0)
13	19.9	70.4	121.0 (99.5–178.0)
13.1	24.3	73.8	123.2 (92.0–380.0)
14	8.1	42.8	77.5 (69.0–91.0)
15	24.9	73.4	121.9 (103.0–160.0)
15.1	24.5	72.4	120.3 (112.5–133.0)
16	5.3	52.2	109.6 (88.0–184.0)
17	3.0	51.1	99.3 (82.0–140.0)
18	6.3	45.9	98.1 (77.0–163.0)

* CI, confidence interval.

### Comparison of window-based and performance-based estimation of incident infection rates

**Figure 2:**
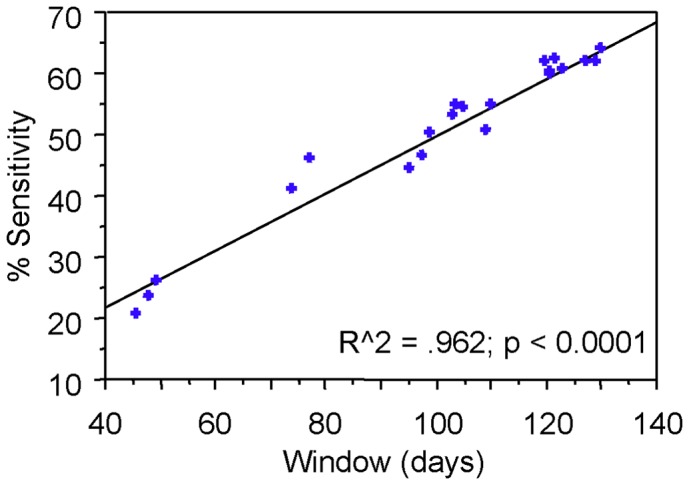
Correlation of window length and diagnostic sensitivity of the algorithms. The diagnostic sensitivity data represent the uncorrected raw sensitivity S_0_, as determined in [19].

We next compared the window periods of the algorithms with their previously determined diagnostic sensitivity [19]. As to be expected, algorithms with a short window (e.g. Algs 3, 3.1, 5) exhibited a low diagnostic sensitivity for detecting infections of up to 12 months duration, while those with long windows were more sensitive (Fig. 2), and there was a good correlation between the two parameters (R^2^ = 0.962; P<0.0001). For further evaluation, we calculated the IIR-W in four annual cohorts of HIV-1 notifications in which we had previously determined the IIR-P based on the performance of the 10 best-performing algorithms [19].

The IIR-W calculated for the first of these cohorts (A, 2005/6) for all 25 algorithms of Table 2 according to Methods, are shown in Table 3. Shown as a reference at the top of the table is the IIR of this cohort, as determined previously based on the BED incidence EIA [17]. Among the total of 748 notifications, the Inno-Lia algorithms ruled between a minimum of 39 cases (Alg3) and a maximum of 151 cases (Alg4.1) as incident, compared to as many as 262 ruled incident by the BED assay. While the number of cases classified as incident thus varied widely between the different Inno-Lia algorithms, exhibiting a coefficient of variation (CV) as high as 30.8%, the number of cases estimated incident and the IIR-W varied considerably less (CV, 12.4 %). This was due to the compensating effect of window length in the equation used for IIR-W estimation (see Methods). The raw IIR-W extended from 0.368 for Alg18 to 0.611 for Alg6, exhibiting a mean of 0.479 (95% CI 0.456–0.520), while the raw IIR derived from the BED assay was 0.836. After adjustment for each algorithm's diagnostic long-term specificity among infections of >12 months duration, as determined in [19], the definite, adjusted IIR-W extended from 0.362 to 0.555 and showed a mean of 0.457 (95% CI 0.438–0.475). In comparison, the adjusted IIR-W for the BED incidence EIA was 0.669. Thus, the mean IIR-W of the Inno-Lia algorithms was 32% lower than the BED-derived IIR-W. Individual 95% CI for the adjusted IIR-W of this cohort A by all 25 algorithms are shown in columns AB and AC of Supporting Material S2.

**Table 3:** Window-based incident infection rates (IIR) among the 748 HIV notifications July 05–June 06.

ALG #	Window days	N ruled incident 1)	N estimated incident	Raw IIR-W	Diagnostic specificity %1)	Adjusted IIR-W
BED-EIA 2)	153	262	625	0.836	*80.1*	0.669
2	103.8	105	369	0.494	*95.4*	0.471
3	45.8	39	311	0.416	*100.0*	0.416
3.1	48.2	44	333	0.445	*100.0*	0.445
3.2	95.4	84	322	0.430	*98.1*	0.422
4	121.0	130	392	0.524	*92.7*	0.486
4.1	130.1	151	424	0.566	*91.9*	0.521
5	49.7	45	330	0.442	*95.5*	0.422
6	74.2	93	457	0.611	*90.8*	0.555
7	105.3	92	319	0.426	*98.4*	0.419
8	110.3	95	314	0.420	*96.8*	0.407
8.1	110.3	94	311	0.416	*96.8*	0.402
9	103.6	88	310	0.414	*98.4*	0.408
11	127.4	128	367	0.490	*93.4*	0.458
11.1	127.4	128	367	0.490	*93.4*	0.458
11.2	127.4	127	364	0.486	*94.1*	0.457
12	129.3	130	367	0.491	*93.4*	0.458
12.1	129.3	130	367	0.491	*93.4*	0.458
13	121.0	123	371	0.496	*95.0*	0.471
13.1	123.2	140	415	0.554	*93.9*	0.521
14	77.5	73	344	0.460	*97.8*	0.450
15	121.9	140	419	0.560	*94.3*	0.529
15.1	120.3	137	416	0.556	*95.1*	0.529
16	109.6	109	363	0.485	*96.6*	0.469
17	99.3	89	327	0.438	*98.2*	0.430
18	98.1	74	275	0.368	*98.2*	0.362
mean	104.4	103.5	358.1	0.479	95.7	0.457
SD	26.2	31.9	44.3	0.059	2.5	0.047
CV%	25.1	30.8	12.4	12.4	2.7	10.3
lower limit 95% CI	94.1	91.0	340.8	0.456	94.7	0.438
upper limit 95% CI	114.6	116.0	375.5	0.502	96.7	0.475

1) Representing the specificity in infections >12 months; taken with permission from [19].

2) BED data taken with permission from [17].

SD, standard deviation; CV%, coefficient of variation shown as percentage; CI, confidence interval.

**Figure 3:**
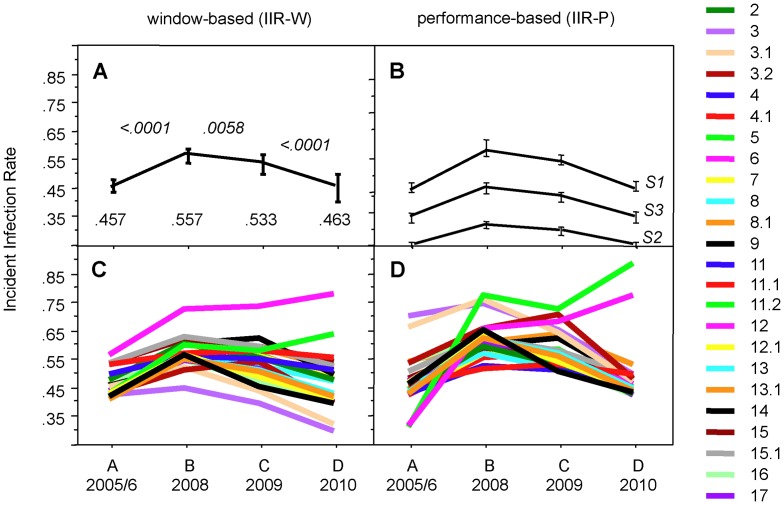
Comparison of window-based and performance-based incident infection rates (IIR) in four annual cohorts of HIV-1 notifications. A) Mean IIR-W and their 95% confidence intervals (CI) of the 25 algorithms of Table 3. The numbers at the bottom of the panel indicate the means of the IIR, numbers in italics on top of the curves denote the P values for the differences according to t-test. B) Mean IIR-P and their 95% CI derived from the 10 best-performing algorithms (Algs 4.1, 7, 8.1, 9, 11.1, 11.2, 12.1, 13, 15, 15.1), as determined in [19]. Shown are the IIR-P curves of three models calculated with diagnostic sensitivities S_1_, S_2_, and S_3_, as defined under Methods; see also Supporting Material S3. C) Individual IIR-W of all 25 algorithms. D) Individual IIR-P of all 25 algorithms based on the diagnostic sensitivities S_1_.

We next assessed the changes over time of IIR-W in four annual cohorts of HIV-1 notifications to the SFOPH (Fig. 3; for full data see columns AA–AU of Supporting Material S2). The first of these cohorts, A (baseline), included the patients of Table 3. Cohorts B, C and D corresponded to the notifications of 2008, 2009 and 2010. All four cohorts had been used previously to assess the IIR using various performance-based approaches (IIR-P) [19].

The mean IIR-W of the 25 algorithms increased from 0.457 in cohort A to 0.557 in cohort B, which meant an increase by 22.4%, a difference highly significant by paired t-test (Fig. 3, panel A). The IIR-W of the 25 algorithms increased individually by a minimum of 6.4% to a maximum of 39.6% (columns AH and AI of Supporting Material S2). For 13 of the 25 algorithms, this initial rise in IIR-W was significant, as shown by the fact that the IIR-W of 2008 exceeded the upper limit of the 95% CI of the respective IIR-W at baseline (see columns AC and AH of Supporting Material S2). In cohort C (2009), the mean IIR-W dropped slightly to 0.533. For 10 of the algorithms, the individual IIR-W levels were still significantly higher than at baseline. In cohort D, the mean IIR-W dropped back to 0.463, which was close to baseline.

When using the algorithms with a performance-based mode of evaluation (see Methods), the resulting IIR-P curves, shown in panel B of Fig. 3, depended strongly on how the diagnostic sensitivity was determined, i.e. whether and how potential selection bias had been handled [19]. Such bias is exerted by the fact that many patients diagnosed with incident HIV-1 infection seek clarification of their HIV status early after exposure, particularly if they exhibit symptoms of an acute retroviral syndrome. This influences the empirically determined diagnostic sensitivity and should be adjusted for. Three different diagnostic sensitivities, S_1_, S_2_ and S_3_ (see Methods), were used in parallel in order to calculate the IIR-P for the 10 algorithms that had performed best in distinguishing incident from older infections [19]; full data are shown in Supporting Material S3.

With sensitivities S_1_, a mean IIR-P of 0.453 was obtained for cohort A. This was near-identical with the window-based IIR-W of 0.457. Between the four cohorts, the curves for IIR-W (Fig. 3A) and IIR-P (Fig. 3B) had similar shapes. The annual changes were more pronounced for IIR-P, however, than for IIR-W. The mean IIR-P showed a steeper initial increase for 2008 (+30.6%) than did the mean IIR-W (+22.4%), but during 2009 and 2010 it also dropped back to baseline levels. Diagnostic sensitivities S_2_ and S_3_ yielded IIR-P curves that were shifted to lower levels compared to S_1_, while maintaining the same relative changes between the four cohorts (see Supporting Material S3). Thus, the IIR-W corresponded best to an IIR-P that was based on the adjusted, but now weighted sensitivities S_1_.

The sensitivities S_1_ were therefore used for an individual comparison of the IIR-W and IIR-P of all 25 algorithms (Fig. 3C and 3D; for full IIR-W data see columns AA to AU of Supporting Material S2). A first glance reveals a distinct initial increase followed by a slow return to baseline as the general trend of the curves. Two algorithms, Alg5 and Alg6, which interprete the antibodies to Gag antigens p24 and p17, did not follow this trend, but continued to increase in cohorts C and D with regard to both IIR-W and IIR-P. The IIR-W and IIR-P of other Algs including 3.2, 4.1, 6, 11, 11.1, 11.2, 12.1, 13.1 and 14 cumulated in cohort C rather than B, and Algs 3 and 3.1 showed a final decrease in IIR well below the baseline. Thus, there was considerable variation among the individual curves for both IIR-W and IIR-P, although the mean IIR of both methods yielded similar results.

## Discussion

The principal goal of this study was to determine the window periods of the more than 20 Inno-Lia algorithms developed previously for estimating the proportion of incident infections in cohorts of HIV-1 infected patients [17]–[19]. Window periods of the different algorithms were determined in a group of 527 patients with incident HIV-1 infection of known duration (Table 1), using linear regression of the proportion of cases ruled incident in dependence of time since infection (Fig. 1) for determining the time-point at which 100% of cases would be classified as older infection (Table 2). Based on these windows, which correlated well with the previously determined diagnostic sensitivity of the respective algorithms (Fig. 2), we calculated the IIR-W for a cohort of HIV notifications for which results of the BED Incidence EIA were available [17]. We found that, on average, Inno-Lia based IIR-W were one-third lower than the IIR-W derived from the BED assay (Table 3). In comparison of four subsequent annual cohorts of HIV notifications we further found that the mean annual IIR-W changes between the four cohorts were similar to those of IIR-P, provided that calculation of the latter was based on a diagnostic sensitivity S_1_ which, like the IIR-W, did not adjust for selection bias (Fig. 3).

The model which yielded sensitivity S_1_ [19] assumed that patients with incident HIV-1 infection would be diagnosed at similar frequency throughout the 12-months incident infection period. This assumption is probably incorrect, as many HIV-exposed patients, especially when experiencing symptoms of acute HIV disease, seek early clarification of their HIV status. When the IIR-P was adjusted for these biases by using the diagnostic sensitivities S_2_ or S_3_ (see Methods), the resulting IIR-P curves were markedly lower than the IIR-W curve (Fig. 3B). Thus, the Inno-Lia based IIR-W, which involves no adjustment for selection bias, corresponded best to the S_1_-based IIR-P, which neither adjusted for such bias. That the two methods exhibit such good agreement is remarkable. Nevertheless, the true IIR is probably lower.

One major advantage of Inno-Lia based IIR estimation is the availability of a whole panel of algorithms, each with its own window length. In contrast to the BED-EIA or other examples of TRIs, Inno-Lia provides a whole panel of tools for assessing each specimen. Thus, all 25 algorithms yielded an increase in the IIR of 2008 compared to 2005/06. Nevertheless, there is considerable variation in the IIR curves of the individual algorithms as demonstrated in panels C and D of Fig. 3. The variation remains high even after removal of Alg5 and Alg6, which are unsuitable for IIR estimation because the underlying antibodies to p24 and p17 are down-regulated early in patients with disease progression [29]–[32], thereby lowering the specificity of these algorithms. We have pointed out previously that it would be impossible to select a “best curve” from those displayed in Fig. 3, and the reliability of both the IIR-W and IIR-P estimates derives from the combination of different algorithms [19]. How the algorithms are best combined remains to be seen. It would make sense to combine the same algorithms for window-based IIR as those that worked best in the performance-based approach [19]; see also Supporting Material S3. Using these, the mean IIR-W for cohorts A, B, C and D amounted to 0.465, 0.564, 0.534 and 0.467, while using the 10 most specific algorithms (according to the long-term specificities of Table 3) yielded 0.416, 0.529, 0.488 and 0.388. Using only the three primary algorithms, Algs 2, 3.1 and 4, which measure the antibody response to gp120, gp41 and respectively p31 and are truly independent of each other, yielded 0.467, 0.553, 0.507, and 0.428. All these curves are similar in shape, and how the algorithms are combined may thus not be that important.

Combination of algorithms is also valuable when assessing differences between annual cohorts of HIV notifications. It should be considered, though, that most of the algorithms are not truly independent of each other (see the Supporting Material S1 for definitions). Comparison of their means by t-test should therefore be done with caution. More than half of the algorithms showed individually significant increases, though, as shown by the fact that the IIR-W of cohort B were above the 95% CI of cohort A (see Supporting Material S2). Moreover, when using the combination of the three independent algorithms 2, 3.1 and 4, their mean IIR-W rose significantly for cohort B compared to A. Thus, both the rise and fall of the IIR-W in the four notification cohorts are confirmed.

The use of an HIV confirmation assay for IIR estimation is a further advantage because, with a well organized national HIV confirmation strategy, near-complete Inno-Lia data can be obtained. In Switzerland, federal regulations issued in 2006 by the SFOPH [33] request that all newly diagnosed cases of HIV infection are tested by the Inno-Lia. This test is valuable for confirming HIV infection and for differentiating between HIV-1 and HIV-2 [27], [28]. Timely diagnosis of HIV-2 infection is important, because HIV-2 requires different viral load tests than the widely used and FDA-approved tests for HIV-1 RNA quantification from Roche, Abbott, BioMérieux, or Bayer. Neither a positive, nor a negative result of these viral load tests excludes an HIV-2 infection. Importantly, treatment of HIV-2 requires different antiretroviral drug regimens, as the virus is naturally resistant to some frequently used drugs including the whole class of non-nucleoside reverse transcriptase inhibitors (NNRTI) [34]–[37].

In Switzerland, HIV confirmation is organized in such a way that detailed Inno-Lia results and other data important in the context of HIV diagnosis and confirmation are collected by 11 regional HIV notification labs and reported by e-mail to the SFOPH using a dedicated Excel® based form. At the SFOPH, the data are linked with supplemental information obtained from the patient's treating physician, archived and evaluated at regular intervals. This schedule works very well, and in this way it has become possible to receive Inno-Lia data for a very high proportion of the new HIV diagnoses. For example, in 2010, Inno-Lia data were available for 99.3% and in 2011 for 555 of the 556 newly diagnosed and notified patients (99.8%). Therefore, the IIR estimates can be considered representative. Inno-Lia based IIR estimation does not require additional tests, nor is shipping of samples to a central lab required. Whether one uses a single algorithm or a combination of different ones has no effect on costs, as these population-based evaluations, once set up, can be done in an automated way, e.g. by pasting the annual Inno-Lia dataset into a simple, pre-formed evaluation table, e.g. based on the Microsoft Excel® software. Linking Inno-Lia based IIR estimation to the context of prospective, individual confirmation of an HIV diagnosis is of advantage, because newly diagnosed patients are generally ART-naive. Prolonged aviremia due to long-term ART, which has been shown to lower the specificity of Inno-Lia based incident infection algorithms [18], will thus not be present. We do not recommend the Inno-Lia for IIR estimation outside of the context of prospective individual confirmation of newly diagnosed HIV infections. It is also clear that the Inno-Lia, a relatively expensive test, is not affordable to low-income countries.

Regarding window length, the present study allows comparison with the findings of other studies. As shown in Table 2, seroconversion in the Inno-Lia starts with antibodies to gp41 (Alg3), which became detectable (intensity ≥0.5) a median 24.5 days after estimated date of infection. Antibodies to p24 (Alg5) appeared almost as fast with a median of 26.9 days, while the median windows of antibodies to p17 (Alg6) , gp120 (Alg2) and p31 (Alg4) were at 38.7, 55.3 and 70.4 days respectively. The sequence of antibody appearance was the same as in a study based on 8 prospectively followed patients with known date of infection [38]. According to other studies with a high number of cases, the mean seroconversion time of IgM-sensitive HIV 3^rd^ generation screening tests is estimated at 22 days with a 95% CI of 18.5 to 25.5 days [23], [39]. Our 24.5 days median for gp41 antibodies is compatible with these estimates. According to another study, Western blot becomes positive a median 26 days after detection of HIV-1 RNA [40]. Again, our 24.5 days median for gp41 antibodies combined with the 26.9 days median for p24 antibodies, which is equivalent to a definition of WB positivity, is compatible with these findings. Regarding p31 antibodies, their mean window, as estimated by Fiebig et al. [23], amounts to about 100 days, with a wide confidence interval of 58 to 140 days. Our median of 70.4 days for Alg4 is again compatible with that estimate. Thus, the shortest and longest windows of our algorithms are in accordance with published findings.

### Limitations

A possible weakness lies in the relatively imprecise information regarding the duration of the infection in some patients that is inherent to such studies and in the low number of cases available at later time-points of the incident infection period (Fig. 1A). As a result, the windows of some algorithms may be underestimated, while others may be overestimated. Use of several different algorithms will level out the resulting differences in the IIR-W calculations, as shown in Table 3 and Fig. 3. It should also be noted that the estimated dates of infection of the 144 patients originating from the ZPHI were available at a very high accuracy and time-dependent resolution, as verified by additional measures such as viral diversity based on clonal HIV-1 *env* C2-V3-C3 sequences [13], [20], [24].

A systematic under- or overestimation of the time since infection would be another possibility. This would affect all windows in the same way, either by increasing or shortening them by a certain number of days. We have studied the effect of such changes. Shortening the windows increased all IIR-W, increasing them diminished the IIR-W. The relative differences between cohorts A, B, C and D did not change, however (data not shown). Thus, independently of how accurate our window estimates are on an absolute scale, we will obtain the same relative changes between these cohorts. The same effect was also found for IIR-P when changing the diagnostic sensitivity [19]; see also Supporting Material S3, which contains IIR-P calculations based on the three different sensitivities S_1_, S_2_ and S_3_.

A further question relates to the diagnostic sensitivity and specificity of the Inno-Lia algorithms. Regarding sensitivity, we defined our windows in such a way that 100% of the newly infected patients would switch from incident to older infection status within the window period (see Methods), which implies a 100% diagnostic sensitivity. Thus, in contrast to other methods where the window was selected differently, e.g. as the mean or median or the time interval within which the method differentiated best between older and incident infection, there is no need for us to correct the sensitivity for cases that had not switched to older infection status at the closure of the window. All cases with such a delayed conversion to older status can be handled as false-incident, as they exhibit an incident antibody pattern in the period defined as older infection. Cases with a delayed conversion thus affect only the diagnostic specificity, but not the sensitivity. As shown in Table 3, all our IIR values are corrected for their imperfect long-term specificity due to the vaning antibody concentrations seen in advanced disease. However, as the specificities of Table 3 relate to an incident infection period definition of 12 months, the short-term specificity of the algorithms from the closure of the window to the end of these 12 months could possibly differ from the long-term specificity. We have investigated this question by determining the percentage of false-incident cases in this period for the algorithms and comparing them to the long-term specificity. Using the subset of well-characterized patients of the ZPHI study, we found a significantly higher frequency of false-incident cases for Algs 7–9 and 18 by 2×2 table test. For all other algorithms, the short-term specificity was similar to that listed in Table 3. The cases diagnosed in this short interval are probably rare, and the impact of a diverging short-term specificity on the IIR should thus be limited. Furthermore, when combining the algorithms for IIR estimation, the influence of a transiently lower specificity should be minimized even further, as such individual errors are “diluted out” by the majority of the unaffected algorithms (see Fig. 3C and 3D). This should also apply to any other possible weakness of individual algorithms. We therefore recommend again that IIR estimation should be based on combinations of algorithms.

In conclusion, Inno-Lia based estimation of the HIV-1 incident infection rate in populations of newly diagnosed patients can also be based on the window periods of the Inno-Lia algorithms. The IIR-W estimates were similar to Inno-Lia based IIR-P estimates, provided that the latter were not corrected for selection bias with respect to patients who seek early clarification of their HIV status after a suspected exposure. We believe, however, that such corrections would be important, and in this respect the lower IIR-P estimates, particularly that based on the diagnostic sensitivity S_3_, probably better reflect the truth (Fig. 3B). It remains to be seen whether such adjustments can also be made for the IIR-W.

Even without such further correction, the Inno-Lia based IIR-W in one of the cohorts was about one-third lower than that based on the BED EIA, which is important when considering that this widely used test frequently yields unrealistically high incident infection rates and has to be corrected for its well-known imperfect sensitivity and specificity [5], [41]–[45]. Unlike the BED EIA, the specificity of the Inno-Lia algorithms in ART-naïve patients is neither affected by the severity of the immunodeficiency, nor by the genetic diversity of HIV [18]. Therefore, Inno-Lia based assessment of incident infection rates does not require prior exclusion of the patients in an advanced stage of disease. We have demonstrated in a large study of patients predominantly infected with non-B subtypes and circulating recombinant forms (CRF) that the clade of HIV-1 does not influence the incidence result [18]. Technically, the method should thus also be feasible for countries that already use the Inno-Lia, yet have an HIV-1 subtype distribution different from that of Switzerland, where subtype B dominates the newly diagnosed infections with about 60% (as based on the sequences of 2670 new patients entered into the national HIV resistance database from 2009–2012).

In short, Inno-Lia based assessment of incident HIV infection rates can be performed without a need for clinical information other than that the patients are treatment-naïve, a requirement always met when a patient is newly diagnosed with HIV infection and undergoes confirmation with supplemental tests. Inno-Lia based IIR estimation in the context of HIV confirmation represents a free, additional public health benefit of the use of this relatively costly test for confirmation of HIV infection and differentiation between HIV-1 and HIV-2.

## Supporting Information

Supporting Material S1 **Definitions and diagnostic performance of Inno-Lia algorithms for incident HIV-1 infection.**
(PDF)

Supporting Material S2 **IIR-W calculations based on time as a linear entity. Complete data.**
(PDF)

Supporting Material S3 **Changes in the performance-based Incident infection rate among four annual cohorts of HIV-1 notifications using the 10 best algorithms.**
(PDF)
