# Supplementary material for: Simple Estimation of Incident HIV Infection Rates in Notification Cohorts Based on Window Periods of Algorithms for Evaluation of Line-Immunoassay Result Patterns
Source: PLoS One. 2013 Aug 26;8(8):e71662. doi: 10.1371/journal.pone.0071662 (PMC3753319; doi:10.1371/journal.pone.0071662)
Supplement: Supporting Material S1 — Definitions and diagnostic performance of Inno-Lia algorithms for incident HIV-1 infection. (PDF) [file pone.0071662.s001.pdf]

Supporting Information S1 – Definitions and diagnostic performance of Inno-Lia algorithms for incident HIV-1 infection (adapted from [19])

Alg #	Definition	% Sensitivity	% Specificity
2	if sgp120≤1<br>then RECENT<br>else older	54.46	95.41
3	if gp41≤0.5<br>then RECENT<br>else older	20.30	100.0
3.1	if gp41≤1<br>then RECENT<br>else older	23.53	100.0
3.2	if gp41≤2<br>then RECENT<br>else older	44.02	98.11
4	if p31=0<br>then RECENT<br>else older	60.15	92.70
4.1	if p31≤0.5<br>then RECENT<br>else older	63.95	91.89
5	if p24≤0<br>then RECENT<br>else older	26.00	95.54
6	if p17=0<br>then RECENT<br>else older	40.80	90.80
7	if sgp120+gp41+p31≤4<br>then RECENT<br>else older	54.08	98.38
8	if gp41≤0.5<br>OR (sgp120+gp41+p31≤4)<br>OR (sgp120+gp41+p31+p24+p17≤ 6.5)<br>then RECENT<br>else older	54.65	96.75
8.1	if gp41≤0.5<br>OR (sgp120+gp41+p31≤4)<br>OR ((sgp120+gp41+p31+p24+p17≤ 6.5) AND p31≤1)<br>then RECENT<br>else older	54.65	96.76
9	if sgp120+gp41≤4 AND p31=0<br>then RECENT<br>else older	52.75	98.38
10	if p31=0 AND p24 ≥ 2<br>then RECENT<br>else older	30.93	95.27
11	if (sgp120+gp41≤2.5)<br>OR (sgp120+gp41+p31+p24+p17≤6.5)<br>OR (p31=0 AND p24 ≥ 2)<br>then RECENT<br>else older	61.48	93.37
11.1	if (sgp120+gp41≤2.5)<br>OR ((sgp120+gp41+p31+p24+p17≤6.5) AND p31≤1<br>OR (p31=0 AND p24 ≥ 2)<br>then RECENT<br>else older	61.48	93.51

11.2	if (sgp120+gp41≤2.5)<br>OR ((sgp120+gp41+p31+p24+p17≤6.5) AND p31≤1 AND p17≤p24)<br>OR (p31=0 AND p24 ≥ 2)<br>then RECENT<br>else older	61.48	94.05
12	if (p24 ≥ 2 AND p31=0)<br>OR (gp41≤.5)<br>OR (sgp120+gp41+p31≤4<br>OR sgp120+gp41+p31+p24+p17≤6.5)<br>then RECENT<br>else older	61.67	93.37
12.1	if (p24 ≥ 2 AND p31=0)<br>OR (gp41≤.5)<br>OR (sgp120+gp41+p31≤4)<br>OR (p31≤1 AND (sgp120+gp41+p31+p24+p17≤6.5))<br>then RECENT<br>else older	61.67	93.38
13	if (sgp120+gp41≤4 AND p31=0)<br>OR (p31=0 AND p24 ≥ 2)<br>then RECENT<br>else older	59.77	95.00
13.1	if gp41≤2<br>OR (p31=0 AND p24 ≥ 2)<br>then recent<br>else older	60.34	93.92
14	if (sgp120+gp41+p31+p24+p17≤6.5 AND p31≤1)<br>then RECENT<br>else older	45.92	97.84
15	if (sgp120≤1 AND p31≤1)<br>OR (gp41≤2 AND p31≤1)<br>OR (p17 ≥ 2 AND p31=0)<br>OR (p31=0 AND p24 ≥ 2)<br>then RECENT<br>else older	62.24	94.32
15.1	if (sgp120≤1 AND p31≤1 AND p17≤p24)<br>OR (gp41≤2 AND p31≤1 AND p17≤p24)<br>OR (p17 ≥ 2 AND p31=0 AND p17≤p24)<br>OR (p31=0 AND p24 ≥ 2 AND p17≤p24)<br>then RECENT<br>else older	61.67	95.14
16	if (sgp120≤1 AND (p31+p24+p17≤2.5))<br>OR (gp41≤1)<br>OR (p31≤0.5 AND (sgp120+gp41+p24+p17≥15))<br>OR (p24=0 AND gp41≤2)<br>OR (p24 ≥ 3 AND p31=0)<br>then RECENT<br>else older	50.47	96.62
17	if (sgp120 * gp41)≤2<br>then RECENT<br>else older	50.09	98.24
18	if (sgp120 * gp41≤1)<br>OR (p24+p31=0)<br>then RECENT<br>else older	46.11	98.24
