# Supplementary material for: Simple Estimation of Incident HIV Infection Rates in Notification Cohorts Based on Window Periods of Algorithms for Evaluation of Line-Immunoassay Result Patterns
Source: PLoS One. 2013 Aug 26;8(8):e71662. doi: 10.1371/journal.pone.0071662 (PMC3753319; doi:10.1371/journal.pone.0071662)
Supplement: Supporting Material S2 — IIR-W calculations based on time as a linear entity. Complete data. (PDF) [file pone.0071662.s002.pdf]

Supporting Information S2: IIR-W calculations based on time as a linear entity

Calculations relating to Table 2 of the paper

Row			Linear regression curve parameters (y = ax + b)		Full window (days)			50% window (days)			0% window (days)			Regression curve specifications		
8		Algorithm	b	a	x for y=0%	Lower Limit 95%CI	Upper Limit 95%CI	x for y=50%	Lower Limit 95%CI	Upper Limit 95%CI	x for y=100%	Lower Limit 95%CI	Uper Limit 95%CI	Datapoints selected	N datapoints	R^2
9		BED			153.0											
10	1	2	107.071	-1.032	103.8	92.0	121.7	55.3	49.5	62.5	6.9	0.0	15.0	3-6	4	0.933
11	2	3	107.456	-2.348	45.8	33.0	114.0	24.5	0.0	39.5	3.2	0.0	17.5	1-4	4	0.938
12	3	3.1	108.831	-2.257	48.2	36.0	94.0	26.1	10.0	38.0	3.9	0.0	16.0	1-4	4	0.953
13	4	3.2	94.406	-0.990	95.4	67.0	85.0	44.9	37.0	44.5	5.7	1.0	11.0	2-6	5	0.963
14	5	4	119.663	-0.989	121.0	99.0	177.5	70.4	62.0	86.0	19.9	0.0	34.5	3-6	4	0.977
15	6	4.1	123.961	-0.953	130.1	94.0	570.0	77.6	62.0	204.0	25.1	0.0	44.5	3-6	4	0.926
16	7	5	109.067	-2.193	49.7	38.0	86.0	26.9	14.0	37.0	4.1	0.0	16.5	1-4	4	0.961
17	8	6	104.317	-1.405	74.2	63.0	96.0	38.7	32.0	46.0	3.1	0.0	14.0	1-5	5	0.963
18	9	7	113.098	-1.074	105.3	92.0	133.0	58.8	52.0	65.5	12.2	0.0	26.0	3-6	4	0.987
19	10	8	109.320	-0.991	110.3	90.0	176.0	59.9	48.0	73.0	9.4	0.0	28.5	3-6	4	0.968
20	11	8.1	109.320	-0.991	110.3	90.0	176.0	59.9	48.0	73.0	9.4	0.0	28.5	3-6	4	0.968
21	12	9	108.484	-1.047	103.6	80.0	250.0	55.9	32.0	74.0	8.1	0.0	32.5	3-6	4	0.941
22	13	11	120.440	-0.945	127.4	109.0	162.0	74.5	67.0	74.0	21.6	5.5	32.5	3-6	4	0.989
23	14	11.1	120.440	-0.945	127.4	109.0	162.0	74.5	67.0	74.0	21.6	5.5	32.5	3-6	4	0.989
24	15	11.2	120.440	-0.945	127.4	109.0	162.0	74.5	67.0	74.0	21.6	5.5	32.5	3-6	4	0.989
25	16	12	120.500	-0.932	129.3	106.0	185.0	75.6	66.0	93.0	22.0	0.0	35.0	3-6	4	0.980
26	17	12.1	120.500	-0.932	129.3	106.0	186.0	75.6	66.0	93.0	22.0	0.0	35.0	3-6	4	0.980
27	18	13	119.663	-0.989	121.0	99.5	178.0	70.4	62.5	86.0	19.9	0.0	34.5	3-6	4	0.977
28	19	13.1	124.596	-1.011	123.2	92.0	380.0	73.8	60.0	140.0	24.3	0.0	43.0	3-6	4	0.936
29	20	14	111.680	-1.441	77.5	69.0	91.0	42.8	37.0	47.0	8.1	0.0	16.0	2-5	4	0.992
30	21	15	125.690	-1.031	121.9	103.0	160.0	73.4	66.0	85.0	24.9	5.0	35.0	3-6	4	0.986
31	22	15.1	125.629	-1.044	120.3	112.5	133.0	72.4	69.0	75.0	24.5	18.0	30.0	3-6	4	0.997
32	23	16	95.383	-0.870	109.6	88.0	184.0	52.2	35.0	63.0	5.3	0.0	19.5	3-6	4	0.965
33	24	17	103.129	-1.039	99.3	82.0	140.0	51.1	40.0	62.5	3.0	0.0	20.0	2-6	5	0.943
34	25	18	93.987	-0.958	98.1	77.0	163.0	45.9	28.0	59.0	6.3	0.0	17.0	2-6	5	0.912
35		*mean*	*112.7*	*-1.2*	*104.4*			*58.2*			*13.4*					*0.965*
36		*SD*	*9.7*	*0.4*	*26.2*			*17.0*			*8.5*					*0.024*
37		*%CV*	*8.6*	*-36.8*	*25.1*			*29.3*			*63.6*					*2.4*
38		*lower limit 95%CI*	*108.9*	*-1.3*	*94.1*			*51.5*			*10.1*					*0.955*
39		*upper limit 95%CI*	*116.5*	*-1.0*	*114.6*			*64.9*			*16.8*					*0.974*

	Q	R	S	T	U	V	W	X	Y	Z	AA	AB	AC	AD	AE	AF	AG	AH	AI
1																			
2											IIR-W calculations relating to Fig. 3 of the paper. IIR-W figures in red indicate a significant difference com								
3																			
4		**Calculations relating to Table 3 of the paper**																	
5																			
6					Cohort A; Baseline; June 2005 — May 2006; N = 748										Cohort B; 2008; N = 667				
7					Nonadjusted IIR-W Calculation						IIR-W adjusted for specificity								
8		ALG #	Specificity (%)		N ruled older	N ruled incident	N estimated incident	Lower Limit 95%CI	Upper Limit 95%CI	Raw IIR-W	Adjusted IIR-W	Lower Limit 95%CI	Upper Limit 95%CI		N ruled older	N ruled incident	N estimated incident	Adjusted IIR-W	IIR-W, in % of baseline
9		BED	80.1		486	262	625			0.836	0.669								
10		2	95.4		643	105	369	315	417	0.494	0.471	0.402	0.531		549	118	396	0.594	126.0
11		3	100.0		709	39	311	125	431	0.416	0.416	0.167	0.577		630	37	295	0.442	106.4
12		3.1	100.0		704	44	333	171	446	0.445	0.445	0.228	0.596		622	45	341	0.511	114.7
13		3.2	98.1		664	84	322	361	458	0.430	0.422	0.473	0.600		578	89	334	0.501	118.8
14		4	92.7		618	130	392	267	479	0.524	0.486	0.331	0.594		535	132	369	0.553	113.9
15		4.1	91.9		597	151	424	97	586	0.566	0.521	0.119	0.720		522	145	374	0.561	107.7
16		5	95.5		703	45	330	191	432	0.442	0.422	0.244	0.552		611	56	393	0.589	139.6
17		6	90.8		655	93	457	354	539	0.611	0.555	0.429	0.654		560	107	478	0.716	129.0
18		7	98.4		656	92	319	252	365	0.426	0.419	0.332	0.480		558	109	372	0.557	132.9
19		8	96.8		653	95	314	197	385	0.420	0.407	0.255	0.498		554	113	362	0.542	133.4
20		8.1	96.8		654	94	311	195	381	0.416	0.402	0.252	0.493		554	113	362	0.542	134.8
21		9	98.4		660	88	310	128	402	0.414	0.408	0.169	0.528		560	107	371	0.556	136.4
22		11	93.4		620	128	367	288	429	0.490	0.458	0.360	0.535		531	136	364	0.545	119.2
23		11.1	93.4		620	128	367	288	429	0.490	0.458	0.360	0.535		531	136	364	0.545	119.2
24		11.2	94.1		621	127	364	286	425	0.486	0.457	0.360	0.535		531	136	366	0.549	120.1
25		12	93.4		618	130	367	256	448	0.491	0.458	0.320	0.559		528	139	366	0.549	119.9
26		12.1	93.4		618	130	367	255	448	0.491	0.458	0.318	0.559		528	139	366	0.549	119.9
27		13	95.0		625	123	371	252	451	0.496	0.471	0.320	0.573		538	129	370	0.554	117.6
28		13.1	93.9		608	140	415	134	555	0.554	0.521	0.169	0.697		532	135	376	0.563	108.1
29		14	97.8		675	73	344	293	386	0.460	0.450	0.383	0.505		581	86	396	0.594	132.1
30		15	94.3		608	140	419	319	496	0.560	0.529	0.403	0.626		523	144	407	0.610	115.3
31		15.1	95.1		611	137	416	376	444	0.556	0.529	0.478	0.565		524	143	413	0.619	117.1
32		16	96.6		639	109	363	216	452	0.485	0.469	0.279	0.584		556	111	357	0.535	114.2
33		17	98.2		659	89	327	232	396	0.438	0.430	0.305	0.520		568	99	358	0.536	124.7
34		18	98.2		674	74	275	166	351	0.368	0.362	0.218	0.461		575	92	336	0.504	139.4
35		*mean*	*95.7*			*103.5*	*358.1*	*240.6*	*441.3*	*0.479*	*0.457*	*0.307*	*0.563*		*mean*	*111.8*	*371.3*	*0.557*	*122.4*
36		*SD*	*2.5*			*31.9*	*44.3*	*77.4*	*56.8*	*0.059*	*0.047*	*0.097*	*0.063*		*SD*	*30.7*	*33.0*	*0.049*	*9.9*
37		*%CV*	*2.7*			*30.8*	*12.4*	*32.1*	*12.9*	*12.4*	*10.3*	*31.4*	*11.2*		*%CV*	*27.4*	*8.9*	*8.9*	*8.1*
38		*lower limit 95%CI*	*94.7*			*91.0*	*340.8*	*210.3*	*419.0*	*0.456*	*0.438*	*0.269*	*0.538*		*lower limit 95%CI*	*99.8*	*358.4*	*0.537*	*118.5*
39		*upper limit 95%CI*	*96.7*			*116.0*	*375.5*	*271.0*	*463.5*	*0.502*	*0.475*	*0.345*	*0.588*		*upper limit 95%CI*	*123.9*	*384.3*	*0.576*	*126.3*

	AJ	AK	AL	AM	AN	AO	AP	AQ	AR	AS	AT	AU
1												
2	**pared to the baseline IIR-W shown in column AA**											
3												
4												
5												
6		**Cohort C, 2009; N = 578**						**Cohort D; 2010; N = 602**				
7												
8		**N ruled older**	**N ruled incident**	**N estimated incident**	**Adjusted IIR-W**	**IIR-W, in % of baseline**		**N ruled older**	**N ruled incident**	**N estimated incident**	**Adjusted IIR-W**	**IIR-W, in % of baseline**
9												
10		**484**	**94**	**316**	**0.546**	**115.9**		**518**	**84**	**282**	**0.468**	**99.4**
11		**550**	**28**	**223**	**0.386**	**92.9**		**580**	**22**	**175**	**0.291**	**70.1**
12		**545**	**33**	**250**	**0.432**	**97.1**		**577**	**25**	**189**	**0.314**	**70.6**
13		**496**	**82**	**308**	**0.533**	**126.3**		**540**	**62**	**233**	**0.387**	**91.7**
14		**466**	**112**	**313**	**0.542**	**111.5**		**494**	**108**	**302**	**0.502**	**103.2**
15		**450**	**128**	**330**	**0.571**	**109.7**		**474**	**128**	**330**	**0.548**	**105.3**
16		**531**	**47**	**330**	**0.570**	**135.2**		**548**	**54**	**379**	**0.629**	**149.1**
17		**484**	**94**	**420**	**0.726**	**130.8**		**498**	**104**	**464**	**0.771**	**138.9**
18		**497**	**81**	**276**	**0.478**	**113.9**		**531**	**71**	**242**	**0.402**	**95.9**
19		**487**	**91**	**291**	**0.504**	**124.0**		**523**	**79**	**253**	**0.420**	**103.3**
20		**488**	**90**	**288**	**0.499**	**123.9**		**525**	**77**	**247**	**0.410**	**101.8**
21		**504**	**74**	**256**	**0.444**	**108.8**		**535**	**67**	**232**	**0.386**	**94.6**
22		**458**	**120**	**321**	**0.555**	**121.3**		**496**	**106**	**283**	**0.471**	**102.9**
23		**458**	**120**	**321**	**0.555**	**121.3**		**496**	**106**	**283**	**0.471**	**102.9**
24		**459**	**119**	**321**	**0.555**	**121.3**		**498**	**104**	**280**	**0.465**	**101.8**
25		**457**	**121**	**319**	**0.552**	**120.5**		**493**	**109**	**287**	**0.477**	**104.2**
26		**457**	**121**	**319**	**0.552**	**120.5**		**493**	**109**	**287**	**0.477**	**104.2**
27		**474**	**104**	**298**	**0.516**	**109.4**		**505**	**97**	**278**	**0.462**	**98.0**
28		**457**	**121**	**337**	**0.582**	**111.8**		**491**	**111**	**309**	**0.513**	**98.5**
29		**501**	**77**	**355**	**0.614**	**136.5**		**538**	**64**	**295**	**0.490**	**108.9**
30		**458**	**120**	**339**	**0.586**	**110.9**		**489**	**113**	**319**	**0.530**	**100.3**
31		**461**	**117**	**338**	**0.584**	**110.5**		**494**	**108**	**312**	**0.518**	**98.0**
32		**495**	**83**	**267**	**0.462**	**98.5**		**529**	**73**	**235**	**0.390**	**83.2**
33		**497**	**81**	**293**	**0.506**	**117.8**		**537**	**65**	**235**	**0.390**	**90.7**
34		**501**	**77**	**281**	**0.487**	**134.7**		**537**	**65**	**238**	**0.395**	**109.1**
35		*mean*	*93.4*	*308.3*	*0.533*	*117.0*		*mean*	*84.4*	*278.8*	*0.463*	*101.1*
36		*SD*	*28.0*	*39.1*	*0.068*	*11.4*		*SD*	*27.6*	*58.9*	*0.098*	*16.4*
37		*%CV*	*30.0*	*12.7*	*12.7*	*9.7*		*%CV*	*32.7*	*21.1*	*21.1*	*16.2*
38		*lower limit 95%CI*	*82.4*	*293.0*	*0.507*	*112.5*		*lower limit 95%CI*	*73.6*	*255.7*	*0.425*	*94.7*
39		*upper limit 95%CI*	*104.4*	*323.7*	*0.560*	*121.5*		*upper limit 95%CI*	*95.3*	*301.9*	*0.501*	*107.5*
