# Supplementary material for: Simple Estimation of Incident HIV Infection Rates in Notification Cohorts Based on Window Periods of Algorithms for Evaluation of Line-Immunoassay Result Patterns
Source: PLoS One. 2013 Aug 26;8(8):e71662. doi: 10.1371/journal.pone.0071662 (PMC3753319; doi:10.1371/journal.pone.0071662)
Supplement: Supporting Material S3 — Changes in the performance-based Incident infection rate among four annual cohorts of HIV-1 notifications using the 10 best algorithms. (PDF) [file pone.0071662.s003.pdf]

Supporting Information S3: Changes in the performance-based incident infection rate (IIR-P) among four annual cohorts of HIV-1 notifications using the 10 best algorithms

A. Calculations based on the adjusted, but not weighted sensitivities S1

	Performance		2005/6 (Baseline); N = 748				2008; N = 667					2009; N = 578					2010; N = 602				
ALG #	Adjusted Sensitivity S1 (%)	Specificity (%)	N ruled older	N ruled incident	N estimated incident	IIR	N ruled older	N ruled incident	N estimated incident	IIR	IIR, in % of baseline	N ruled older	N ruled incident	N estimated incident	IIR	IIR, in % of baseline	N ruled older	N ruled incident	N estimated incident	IIR	IIR, in % of baseline
15.1	31.86	95.14	611	137	373	0.498	524	143	410	0.614	123.2	461	117	329	0.570	114.3	494	108	292	0.484	97.2
15	32.60	94.32	608	140	362	0.484	523	144	394	0.591	122.0	458	120	324	0.560	115.7	489	113	293	0.486	100.4
11.2	31.37	94.05	621	127	324	0.434	531	136	379	0.568	130.9	459	119	333	0.576	132.7	498	104	268	0.446	102.7
7	24.59	98.38	656	92	348	0.465	558	109	427	0.641	137.9	497	81	312	0.540	116.1	531	71	267	0.443	95.3
13	30.03	95.00	625	123	342	0.457	538	129	382	0.573	125.3	474	104	300	0.519	113.5	505	97	267	0.444	97.1
12.1	31.44	93.38	619	129	320	0.428	528	139	382	0.573	133.8	458	120	329	0.570	133.1	495	107	271	0.449	105.0
9	23.74	98.38	660	88	343	0.459	560	107	435	0.652	142.2	504	74	292	0.506	110.2	535	67	259	0.430	93.7
4.1	34.59	91.89	597	151	341	0.456	522	145	343	0.515	112.9	450	128	306	0.530	116.2	474	128	299	0.497	108.9
11.1	31.37	93.37	620	128	317	0.424	531	136	371	0.556	131.3	458	120	330	0.571	134.8	496	106	267	0.444	104.7
8.1	25.17	96.76	654	94	318	0.425	554	113	417	0.625	146.9	488	90	325	0.562	132.2	525	77	262	0.436	102.4
mean	**29.68**	**95.07**		***mean***	**339**	**0.453**		***mean***	**394**	**0.591**	**130.6**		***mean***	**318**	**0.550**	**121.9**		***mean***	**274**	**0.456**	**100.7**
SD	3.77	2.17		*SD*	19	0.025		*SD*	28	0.042	10.2		*SD*	14	0.025	9.9		*SD*	14	0.024	4.9
lower limit 95%CI	*27.34*	*93.72*		*lower limit 95%CI*	*327*	*0.437*		*lower limit 95%CI*	*377*	*0.565*	*124.3*		*lower limit 95%CI*	*309*	*0.535*	*115.7*		*lower limit 95%CI*	*266*	*0.441*	*97.7*
upper limit 95%CI	*32.01*	*96.41*		*upper limit 95%CI*	*351*	*0.469*		*upper limit 95%CI*	*412*	*0.617*	*136.9*		*upper limit 95%CI*	*327*	*0.566*	*128.0*		*upper limit 95%CI*	*283*	*0.471*	*103.7*

B. Calculations based on the adjusted and weighted sensitivities S2

	Performance		2005/6 (Baseline); N = 748				2008; N = 667					2009; N = 578					2010; N = 602				
ALG #	Adjusted Sensitivity S2 (%)	Specificity (%)	N ruled older	N ruled incident	N estimated incident	IIR	N ruled older	N ruled incident	N estimated incident	IIR	IIR, in % of baseline	N ruled older	N ruled incident	N estimated incident	IIR	IIR, in % of baseline	N ruled older	N ruled incident	N estimated incident	IIR	IIR, in % of baseline
15.1	52.94	95.14	611	137	209	0.280	524	143	230	0.345	123.2	461	117	185	0.320	114.3	494	108	164	0.272	97.2
15	53.57	94.32	608	140	204	0.272	523	144	222	0.332	122.0	458	120	182	0.315	115.7	489	113	165	0.273	100.4
11.2	52.60	94.05	621	127	177	0.236	531	136	206	0.310	130.9	459	119	181	0.314	132.7	498	104	146	0.243	102.7
7	45.15	98.38	656	92	184	0.245	558	109	226	0.338	137.9	497	81	165	0.285	116.1	531	71	141	0.234	95.3
13	50.87	95.00	625	123	187	0.249	538	129	209	0.313	125.3	474	104	164	0.283	113.5	505	97	146	0.242	97.1
12.1	52.76	93.38	619	129	172	0.230	528	139	206	0.308	133.8	458	120	177	0.306	133.1	495	107	146	0.242	105.0
9	43.91	98.38	660	88	179	0.240	560	107	227	0.341	142.2	504	74	153	0.264	110.2	535	67	135	0.225	93.7
4.1	55.08	91.89	597	151	192	0.257	522	145	194	0.290	112.9	450	128	173	0.299	116.2	474	128	169	0.280	108.9
11.1	52.60	93.37	620	128	171	0.228	531	136	200	0.299	131.3	458	120	178	0.307	134.8	496	106	144	0.239	104.7
8.1	45.81	96.76	654	94	164	0.219	554	113	215	0.322	146.9	488	90	167	0.290	132.2	525	77	135	0.224	102.4
mean	**50.53**	**95.07**		***mean***	**184**	**0.246**		***mean***	**213**	**0.320**	**130.6**		***mean***	**172**	**0.298**	**121.9**		***mean***	**149**	**0.247**	**100.7**
SD	4.01	2.17		*SD*	15	0.019		*SD*	13	0.019	10.2		*SD*	10	0.018	9.9		*SD*	12	0.020	4.9
lower limit 95%CI	*48.05*	*93.72*		*lower limit 95%CI*	*175*	*0.234*		*lower limit 95%CI*	*206*	*0.308*	*124.3*		*lower limit 95%CI*	*166*	*0.287*	*115.7*		*lower limit 95%CI*	*141*	*0.235*	*97.7*
upper limit 95%CI	*53.01*	*96.41*		*upper limit 95%CI*	*193*	*0.258*		*upper limit 95%CI*	*221*	*0.331*	*136.9*		*upper limit 95%CI*	*179*	*0.309*	*128.0*		*upper limit 95%CI*	*157*	*0.260*	*103.7*

C. Calculations based on the adjusted and weighted sensitivities S3

	Performance		2005/6 (Baseline); N = 748				2008; N = 667					2009; N = 578					2010; N = 602				
ALG #	Adjusted Sensitivity S3 (%)	Specificity (%)	N ruled older	N ruled incident	N estimated incident	IIR	N ruled older	N ruled incident	N estimated incident	IIR	IIR, in % of baseline	N ruled older	N ruled incident	N estimated incident	IIR	IIR, in % of baseline	N ruled older	N ruled incident	N estimated incident	IIR	IIR, in % of baseline
15.1	39.61	95.14	611	137	290	0.387	524	143	318	0.477	123.2	461	117	256	0.443	114.3	494	108	227	0.376	97.2
15	40.22	94.32	608	140	282	0.377	523	144	307	0.461	122.0	458	120	252	0.437	115.7	489	113	228	0.379	100.4
11.2	39.20	94.05	621	127	248	0.332	531	136	290	0.434	130.9	459	119	254	0.440	132.7	498	104	205	0.341	102.7
7	32.38	98.38	656	92	260	0.347	558	109	319	0.479	137.9	497	81	233	0.403	116.1	531	71	199	0.331	95.3
13	37.65	95.00	625	123	262	0.350	538	129	293	0.439	125.3	474	104	230	0.398	113.5	505	97	205	0.340	97.1
12.1	39.30	93.38	619	129	243	0.325	528	139	290	0.435	133.8	458	120	250	0.433	133.1	495	107	205	0.341	105.0
9	31.42	98.38	660	88	255	0.340	560	107	323	0.484	142.2	504	74	217	0.375	110.2	535	67	192	0.319	93.7
4.1	42.05	91.89	597	151	266	0.356	522	145	268	0.402	112.9	450	128	239	0.414	116.2	474	128	233	0.388	108.9
11.1	39.20	93.37	620	128	241	0.322	531	136	282	0.422	131.3	458	120	251	0.434	134.8	496	106	203	0.337	104.7
8.1	33.09	96.76	654	94	234	0.312	554	113	306	0.459	146.9	488	90	239	0.413	132.2	525	77	193	0.320	102.4
mean	**37.41**	**95.07**		***mean***	**258**	**0.345**		***mean***	**300**	**0.449**	**130.6**		***mean***	**242**	**0.419**	**121.9**		***mean***	**209**	**0.347**	**100.7**
SD	3.72	2.17		*SD*	18	0.024		*SD*	18	0.027	10.2		*SD*	13	0.022	9.9		*SD*	15	0.025	4.9
lower limit 95%CI	*35.11*	*93.72*		*lower limit 95%CI*	*247*	*0.330*		*lower limit 95%CI*	*288*	*0.432*	*124.3*		*lower limit 95%CI*	*234*	*0.405*	*115.7*		*lower limit 95%CI*	*200*	*0.332*	*97.7*
upper limit 95%CI	*39.71*	*96.41*		*upper limit 95%CI*	*269*	*0.360*		*upper limit 95%CI*	*311*	*0.466*	*136.9*		*upper limit 95%CI*	*250*	*0.433*	*128.0*		*upper limit 95%CI*	*218*	*0.363*	*103.7*
